# Brazilian university students: predictors of seeking mental health care for a second time

**DOI:** 10.1590/S1516-31802011000300010

**Published:** 2011-05-05

**Authors:** Clarissa de Rosalmeida Dantas, Amilton dos Santos, Maria Lilian Coelho de Oliveira, Renata Cruz Soares de Azevedo, Claudio Eduardo Muller Banzato

**Affiliations:** I MD, MSc. Psychiatrist, Department of Medical Psychology and Psychiatry, School of Medicine, Universidade Estadual de Campinas (Unicamp), Campinas, São Paulo, Brazil.; II MD. Psychiatrist, Department of Medical Psychology and Psychiatry, School of Medicine, Universidade Estadual de Campinas, Campinas, São Paulo, Brazil.; III Psychologist, Students’ Psychiatric and Psychological Care Service, School of Medicine, Universidade Estadual de Campinas, Campinas, São Paulo, Brazil.; IV MD, PhD. Professor, Department of Medical Psychology and Psychiatry, School of Medicine, Universidade Estadual de Campinas, Campinas, São Paulo, Brazil.; V MD, PhD. Adjunct Professor, Department of Medical Psychology and Psychiatry, School of Medicine, Universidade Estadual de Campinas, Campinas, São Paulo, Brazil.

Dear editor,

We have previously described the demographic characteristics and complaints of the 2,914 students (undergraduates and postgraduates) at a Brazilian public university (Universidade Estadual de Campinas, Unicamp) who sought the campus mental health service for students (Serviço de Assistência Psicológica e Psiquiátrica ao Estudante, SAPPE) over a 17-year period (1987-2004).^1^ Among those 2,914 students, 392 (13.5%) sought the service again after completion of their initial treatment, whereas 2,522 (86.5%) had no further contact with the service. It is important to notice that SAPPE has an open-door policy and students are able to receive mental healthcare at the service as many times as they require, throughout the duration of their undergraduate or postgraduate courses. In order to identify predictors of coming back for another period of treatment, we performed multiple logistic regression taking the demographic characteristics and reported complaints that were described in our earlier paper as independent variables.^1^ The results from the multiple logistic regression are presented below in Table 1.

**Table 1:** Predictors for students who seek mental healthcare at the campus service more than once (multiple logistic regression)

	Estimate^*^	Standard error	Odds ratio	95% confidence interval	P value
Intercept	-2.988	0.230	-	-	0.000
Age < 20 years	1.037	0.198	2.819	1.912 ; 4.157	0.000
Humanities and Arts as the study field	0.398	0.190	1.489	1.026 ; 2.163	0.036
Living in the campus halls of residence	0.574	0.134	1.776	1.367 ; 2.307	0.000
Other people living with the family	0.353	0.153	1.423	1.053 ; 1.922	0.021
Having half-siblings	0.384	0.188	1.467	1.015 ; 2.121	0.041
Age of student's mother < 55 years	0.370	0.163	1.447	1.051 ; 1.993	0.024
Complaint of poor memory	0.825	0.385	2.282	1.073 ; 4.855	0.032
Complaint of low self-esteem	0.570	0.216	1.768	1.157 ; 2.701	0.008

* Coefficients of the logistic equation.

Taken together with the findings from our previous study, the results from the multiple logistic regression suggest that there is a student profile that may particularly need support from the campus mental health care service. We reported previously that students who lived in the campus hall of residence were overrepresented among SAPPE's clients compared with the entire university student body,^1^ and now we find that living in the campus halls of residence means that student are almost twice as likely to seek help at SAPPE more than once. Students who apply to live in the limited campus residence facilities at Unicamp are selected on the basis of social and financial criteria.

Our findings suggest that the role of SAPPE as a mental healthcare provider is even more important for students in a worse economic situation. Student-clients’ mean age when they first sought help at SAPPE was 23.3 years (standard deviation, SD = 5.1; median: 22 years),^1^ and being younger than 20 years of age at first contact with the service increases the likelihood of seeking help at SAPPE again by a factor of 2.8 times. On the one hand, if students are younger than 20 years at first contact with the service, it probably means that they are in the first years of the undergraduate course and therefore they have more years ahead at the university in which to seek the service again. On the other hand, seeking the service during the first year of a course might indicate that mental distress is present early on during university life, and perhaps even prior to university entry.

Although poor memory and low self-esteem were not among the ten complaints most frequently reported by student-clients,^1^ presenting one of those complaints at the time of the first contact with the service was associated with seeking the service again later on. One possible interpretation for this finding is that complaints of poor memory at such a young age are a proxy for academic difficulties.

Students who have a worse economic situation experience academic difficulties and low self-esteem early in their undergraduate course and might represent a vulnerable group that is not only more prone to seeking help from the campus mental health service, but also to demanding mental health support at different times throughout their undergraduate and postgraduate courses. Especially in developing countries where financial resources are limited, university mental health services have to constantly adapt their programs in order to meet the student body's needs in a cost-effective way. Identifying risk factors for the need for further assistance by university mental health care services might help in planning preventive mental health programs designed to target student groups with specific needs.
